# Supplementary material for: Central amygdala single-nucleus atlas reveals chromatin and gene transcription dynamics in human alcohol use disorder
Source: Nat Commun. 2026 Jan 19;17:1634. doi: 10.1038/s41467-026-68351-1 (PMC12905162; doi:10.1038/s41467-026-68351-1)
Supplement: Supplementary file 1 — Supplementary Information [file 41467_2026_68351_MOESM1_ESM.pdf]

SUPPLEMENTARY INFORMATION

Central amygdala single-nucleus atlas reveals chromatin and gene transcription dynamics in human alcohol use disorder

Che Yu Lee[1,#], Ahyeon Hwang[1,2,#], Delaney McRiley[3,#], Jaywon Lee[3], Genevieve Thibodeau[3], Catharine Duman[3], Xiangyu Zhang[4], Mario Skarica[3], Jensine Coudriet[3], Siwei Xu[1], Rosemarie Terwilliger[3], Alexa-Nicole Sliby[3], Jiawei Wang[3], Tuan Nguyen[3], Yujing Liu[3], Hongyu Li[4], Yi Dai[1], Ziheng Duan[1], Yutong Lei[1], Yingxin Lin[4] , Jill R. Glausier[6], David A. Lewis[6], Joel Gelernter[3,5], Paul E. Holtzheimer[5,7], Ke Xu[3,6], Hang Zhou[4], Hongyu Zhao[4], Summer Thompson[3], John H. Krystal[3,5], Alicia Che[3], Jane R. Taylor[3], Jing Zhang[1,2,*], Matthew J. Girgenti[3,5,*]

AFFILIATIONS

[1] Department of Computer Science, University of California, Irvine, California, USA.

[2] Mathematical, Computational and Systems Biology Program, University of California, Irvine, California, USA.

[3] Department of Psychiatry, Yale University School of Medicine, 34 Park Street, New Haven, CT 06520, USA.

[4] Department of Biostatistics, Yale University School of Public Health, New Haven, CT 06510, USA.

[5] National Center for PTSD, U.S. Department of Veterans Affairs.

[6] Department of Psychiatry, University of Pittsburgh School of Medicine, Pittsburgh, PA 15213, USA.

[7] Department of Psychiatry, Geisel School of Medicine at Dartmouth, Lebanon, NH 03756, USA

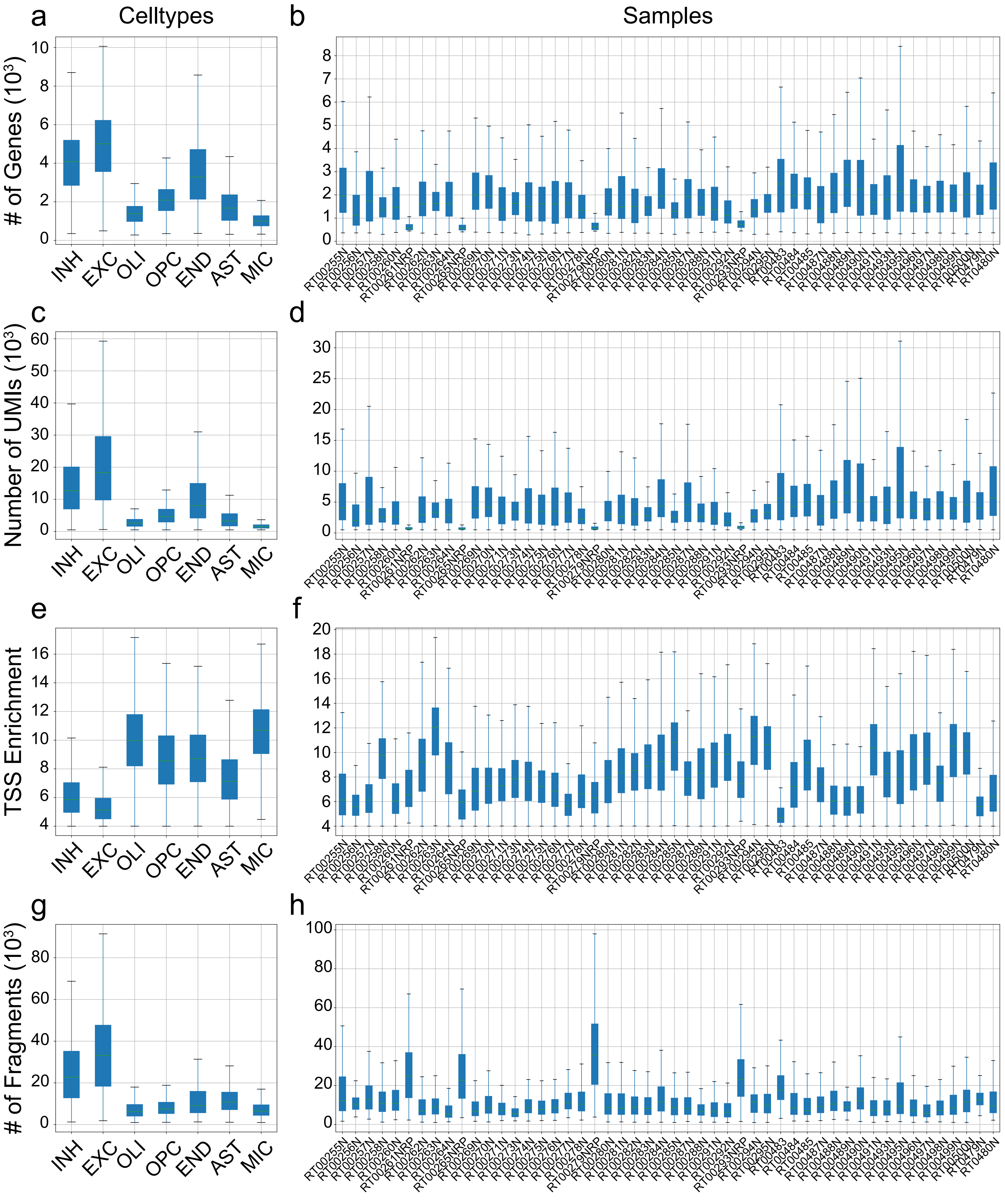
a
Celltypes
b
Samples
# of Genes (10³)
INH
EXC
OLI
OPC
END
AST
MIC
RT00255N
RT00256N
RT00257N
RT00258N
RT00260N
RT00261NRP
RT00262N
RT00263N
RT00264N
RT00265NRP
RT00269N
RT00270N
RT00271N
RT00273N
RT00274N
RT00275N
RT00276N
RT00277N
RT00278N
RT00279NRP
RT00280N
RT00281N
RT00282N
RT00283N
RT00284N
RT00285N
RT00287N
RT00288N
RT00291N
RT00292N
RT00293NRP
RT00294N
RT00295N
RT00483
RT00484
RT00485
RT00487N
RT00488N
RT00489N
RT00490N
RT00491N
RT00493N
RT00495N
RT00496N
RT00497N
RT00498N
RT00499N
RT00500N
RT0479N
RT0480N
c
d
Number of UMIs (10³)
e
f
TSS Enrichment
g
h
# of Fragments (10³)

Supplementary Figure 1: snMultiome sequencing quality control. a, Boxplot of the number of genes across 7 cell types and **b,** 50 samples. **c,** Boxplot of the number of UMIs across 7 cell types and **d,** 50 samples. **e,** Boxplot of TSS Enrichment across 7 cell types and **f,** 50 samples. **g,** Boxplot of the number of fragments across 7 cell types and **h,** 50 samples.

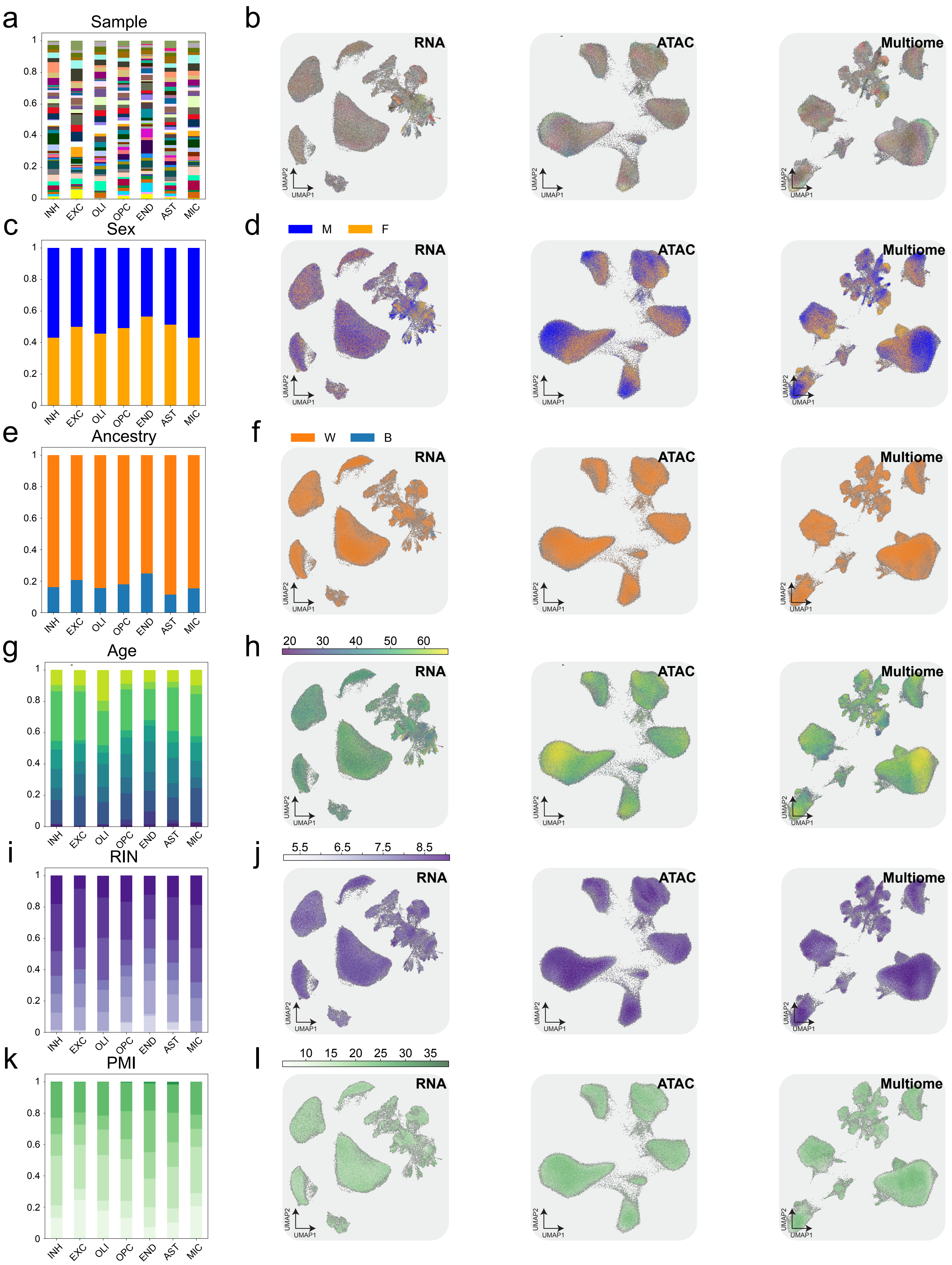

a
Sample
b
RNA
ATAC
Multiome
c
Sex
d
M
F
e
Ancestry
f
W
B
g
Age
h
20 30 40 50 60
i
RIN
j
5.5 6.5 7.5 8.5
k
PMI
l
10 15 20 25 30 35
INH EXC OLI OPC END AST MIC
UMAP1
UMAP2

Supplementary Figure 2: Covariate metadata. a, Sample metadata proportion across 7 cell types and **b,** RNA, ATAC, and Multiome UMAPs. **c,** Sex metadata proportion across 7 cell types and **d,** RNA, ATAC, and Multiome UMAPs. **e,** Ancestry metadata proportion across 7 cell types and **f,** RNA, ATAC, and Multiome UMAPs. **g,** Age metadata proportion of 10 bins across 7 cell types and **h,** RNA, ATAC, and Multiome UMAPs. **i,** RIN metadata proportion of 10 bins across 7 cell types and **j,** RNA, ATAC, and Multiome UMAPs. **k,** PMI metadata proportion of 10 bins across 7 cell types and **l,** RNA, ATAC, and Multiome UMAPs.

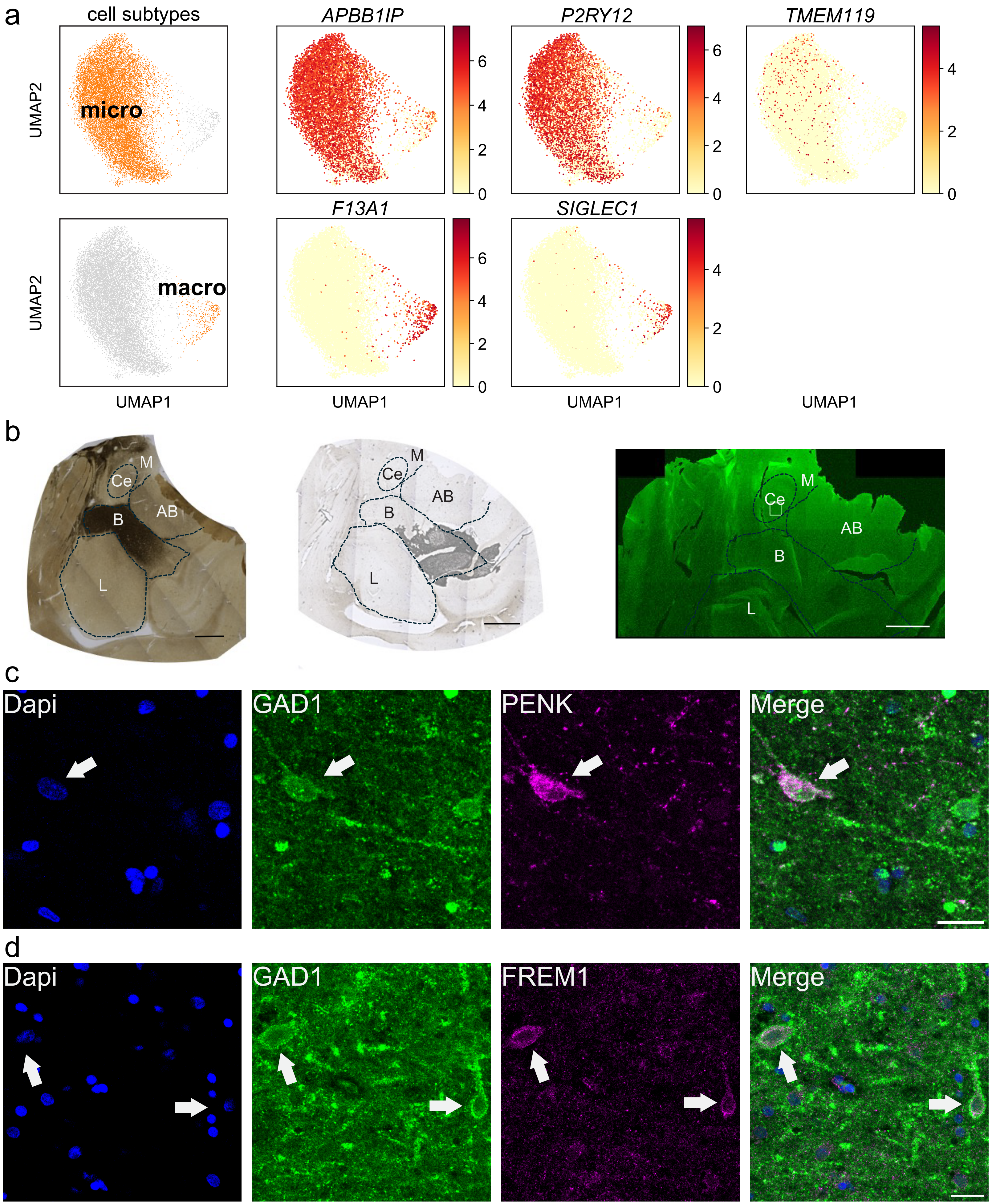

a
cell subtypes
APBB1IP
P2RY12
TMEM119
micro
UMAP2
F13A1
SIGLEC1
macro
UMAP1
b
M
Ce
B
AB
L
c
Dapi
GAD1
PENK
Merge
d
Dapi
GAD1
FREM1
Merge

Supplementary Figure 3: Experimental Validation of Cell subtypes. a, Marker genes used for microglia and macrophage subtypes. **b,** Left: Delineation of amygdala subnuclei is shown by acetylcholinesterase histochemistry in a section adjacent to immuno-labeled sections. High enzymatic activity is evident in the basolateral amygdala, while it is more moderate in the accessory basal nucleus and markedly less in the central and medial nuclei. Middle: brightfield view of the same fluorescent-labeled section shown on the right. Right: fluorescent-labeled section used for marker localization, showing imaging region in Ce (white box). Scale bars 2.5 mm. **c,** An INH neuron in the CeA identified by immunolabel for GAD1 (arrow, second panel), is also PENK immunoreactive. Dapi channel and merge are shown in first and last panels respectively. Scale bar 20 um. **d,** Two GAD1 immunopositive cells in the CeA (arrows, second panel) are co-labeled with FREM1 antibody (arrows, third panel). Dapi channel and merge are shown in first and last panels respectively. Scale bar 20 um. (abbreviations: AB, accessory basal nucleus; B, basolateral nucleus; Ce, central nucleus; L, lateral nucleus; M. medial nucleus).

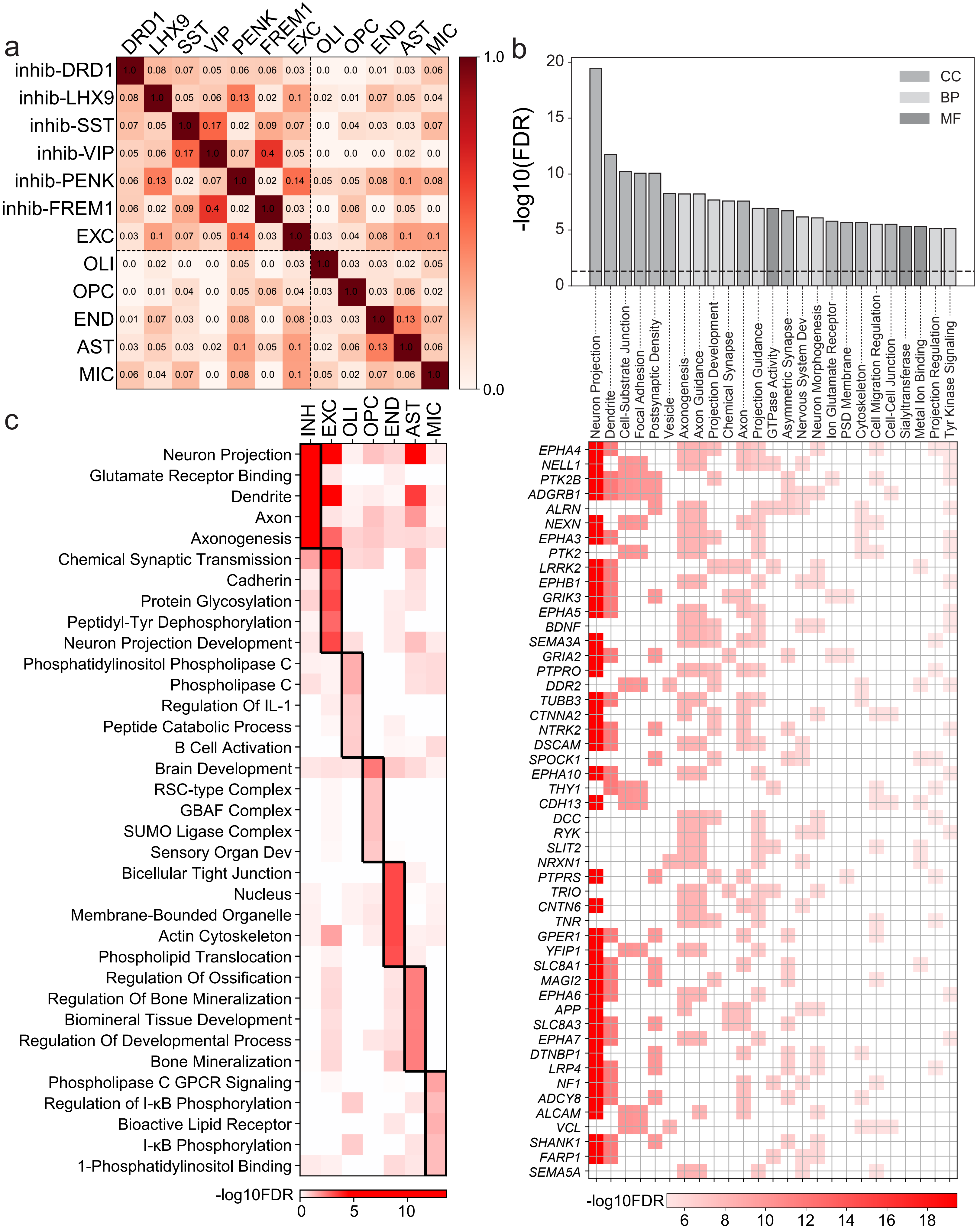
a
DRD1 LHX9 SST VIP PENK FREM1 EXC OLI OPC END AST MIC
inhib-DRD1 1.0 0.08 0.07 0.05 0.06 0.06 0.03 0.0 0.0 0.01 0.03 0.06
inhib-LHX9 0.08 1.0 0.05 0.06 0.13 0.02 0.1 0.02 0.01 0.07 0.05 0.04
inhib-SST 0.07 0.05 1.0 0.17 0.02 0.09 0.07 0.0 0.04 0.03 0.03 0.07
inhib-VIP 0.05 0.06 0.17 1.0 0.07 0.4 0.05 0.0 0.0 0.0 0.02 0.0
inhib-PENK 0.06 0.13 0.02 0.07 1.0 0.02 0.14 0.05 0.05 0.08 0.1 0.08
inhib-FREM1 0.06 0.02 0.09 0.4 0.02 1.0 0.03 0.0 0.06 0.0 0.05 0.0
EXC 0.03 0.1 0.07 0.05 0.14 0.03 1.0 0.03 0.04 0.08 0.1 0.1
OLI 0.0 0.02 0.0 0.0 0.05 0.0 0.03 1.0 0.03 0.03 0.02 0.05
OPC 0.0 0.01 0.04 0.0 0.05 0.06 0.04 0.03 1.0 0.03 0.06 0.02
END 0.01 0.07 0.03 0.0 0.08 0.0 0.08 0.03 0.03 1.0 0.13 0.07
AST 0.03 0.05 0.03 0.02 0.1 0.05 0.1 0.02 0.06 0.13 1.0 0.06
MIC 0.06 0.04 0.07 0.0 0.08 0.0 0.1 0.05 0.02 0.07 0.06 1.0
1.0
0.0
b
-log10(FDR)
20
15
10
5
0
CC
BP
MF
Neuron Projection
Dendrite
Cell-Substrate Junction
Focal Adhesion
Postsynaptic Density
Vesicle
Axonogenesis
Axon Guidance
Projection Development
Chemical Synapse
Axon
Projection Guidance
GTPase Activity
Asymmetric Synapse
Nervous System Dev
Neuron Morphogenesis
Ion Glutamate Receptor
PSD Membrane
Cytoskeleton
Cell Migration Regulation
Cell-Cell Junction
Sialyltransferase
Metal Ion Binding
Projection Regulation
Tyr Kinase Signaling
EPHA4
NELL1
PTK2B
ADGRB1
ALRN
NEXN
EPHA3
PTK2
LRRK2
EPHB1
GRIK3
EPHA5
BDNF
SEMA3A
GRIA2
PTPRO
DDR2
TUBB3
CTNNA2
NTRK2
DSCAM
SPOCK1
EPHA10
THY1
CDH13
DCC
RYK
SLIT2
NRXN1
PTPRS
TRIO
CNTN6
TNR
GPER1
YFIP1
SLC8A1
MAGI2
EPHA6
APP
SLC8A3
EPHA7
DTNBP1
LRP4
NF1
ADCY8
ALCAM
VCL
SHANK1
FARP1
SEMA5A
-log10FDR
6 8 10 12 14 16 18
c
INH EXC OLI OPC END AST MIC
Neuron Projection
Glutamate Receptor Binding
Dendrite
Axon
Axonogenesis
Chemical Synaptic Transmission
Cadherin
Protein Glycosylation
Peptidyl-Tyr Dephosphorylation
Neuron Projection Development
Phosphatidylinositol Phospholipase C
Phospholipase C
Regulation Of IL-1
Peptide Catabolic Process
B Cell Activation
Brain Development
RSC-type Complex
GBAF Complex
SUMO Ligase Complex
Sensory Organ Dev
Bicellular Tight Junction
Nucleus
Membrane-Bounded Organelle
Actin Cytoskeleton
Phospholipid Translocation
Regulation Of Ossification
Regulation Of Bone Mineralization
Biomineral Tissue Development
Regulation Of Developmental Process
Bone Mineralization
Phospholipase C GPCR Signaling
Regulation of I-κB Phosphorylation
Bioactive Lipid Receptor
I-κB Phosphorylation
1-Phosphatidylinositol Binding
-log10FDR
0 5 10

Supplementary Figure 4: Gene Ontology Enrichment of AUD DEGs. a, Cosine similarity heatmap of the cell type DEG lists iInhibitory cell type DEGs were composed from 6 INH sub cell types). Neuronal cell types share high similarity compared to non-neuronal cell types. **b,** Top 25 enrichR GO terms of the 1,805 unique AUD DEG set (Postsynaptic Density is abbreviated as PSD) with the top 50 occurring DEGs. **c,** Cell type-specific enrichR GO terms using cell type-specific DEGs. GO terms were prioritized based on cell type-specificity (Phosphodiesterase is abbreviated as PDE).

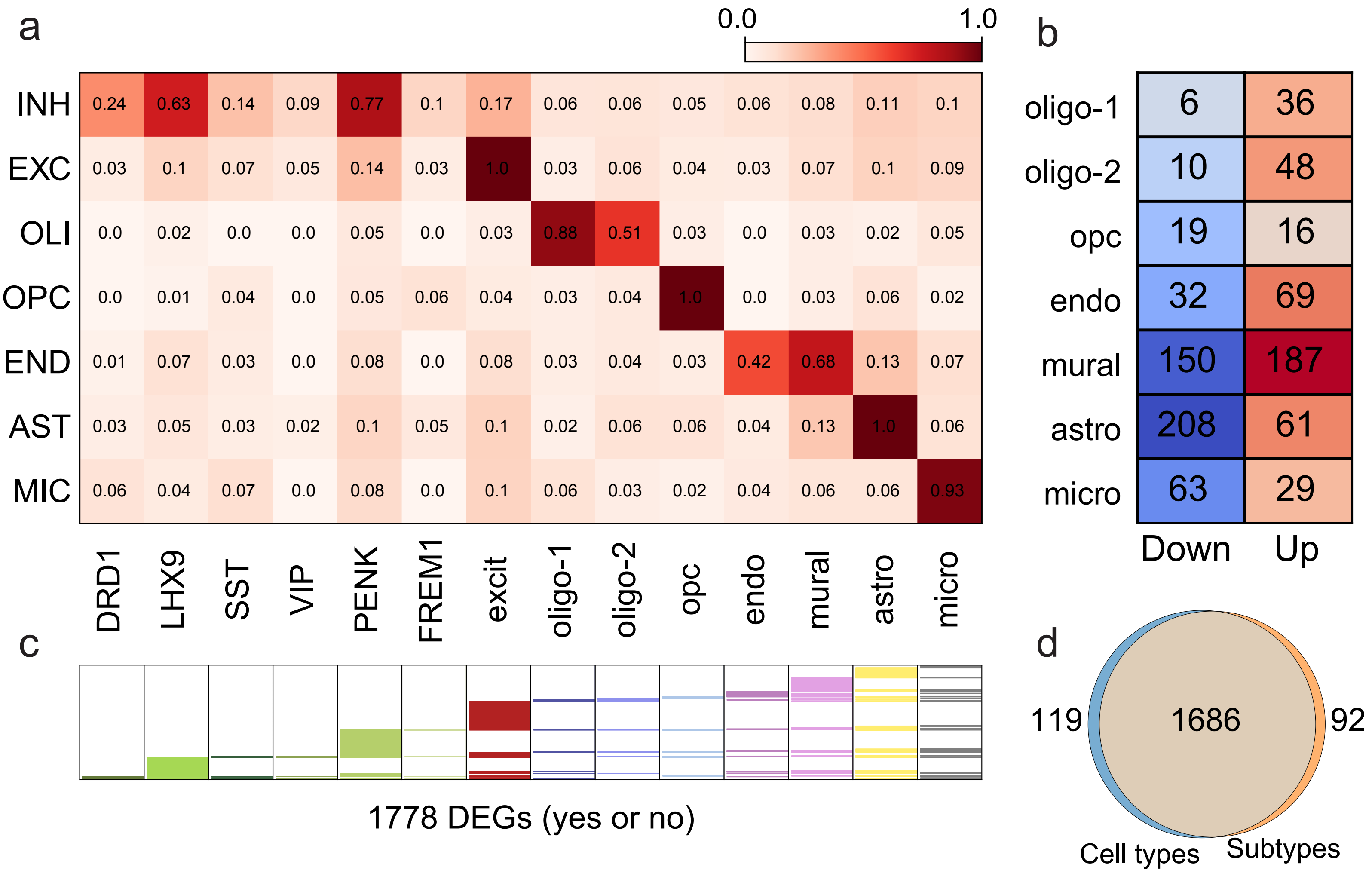
a
0.0
1.0
INH 0.24 0.63 0.14 0.09 0.77 0.1 0.17 0.06 0.06 0.05 0.06 0.08 0.11 0.1
EXC 0.03 0.1 0.07 0.05 0.14 0.03 1.0 0.03 0.06 0.04 0.03 0.07 0.1 0.09
OLI 0.0 0.02 0.0 0.0 0.05 0.0 0.03 0.88 0.51 0.03 0.0 0.03 0.02 0.05
OPC 0.0 0.01 0.04 0.0 0.05 0.06 0.04 0.03 0.04 1.0 0.0 0.03 0.06 0.02
END 0.01 0.07 0.03 0.0 0.08 0.0 0.08 0.03 0.04 0.03 0.42 0.68 0.13 0.07
AST 0.03 0.05 0.03 0.02 0.1 0.05 0.1 0.02 0.06 0.06 0.04 0.13 1.0 0.06
MIC 0.06 0.04 0.07 0.0 0.08 0.0 0.1 0.06 0.03 0.02 0.04 0.06 0.06 0.93
DRD1 LHX9 SST VIP PENK FREM1 excit oligo-1 oligo-2 opc endo mural astro micro
b
oligo-1 6 36
oligo-2 10 48
opc 19 16
endo 32 69
mural 150 187
astro 208 61
micro 63 29
Down Up
c
1778 DEGs (yes or no)
d
119
1686
92
Cell types
Subtypes

Supplementary Figure 5: AUD DEGs at the Subtype level. a, Cosine similarity heatmap of the 7 cell types and 15 sub cell type DEG lists (0 DEGs macrophages). **b,** Significant DEG counts in both directions for each cell subtype. DEGs met a threshold of FC > 1.2 and FDR < 0.01 and overlap between MAST and Wilcox analyses. The intensity of colors (blue downregulated and red upregulated) is proportional to the entry values. **c,** Binary plot indicating whether a gene (row) is a DEG in a given cell subtype (column) or not (n=1,778 unique DEGs from the 15 cell subtypes in **a**). **d,** Venn diagram comparing the 1,805 AUD unique DEGs at the celltype level (left) and the 1,778 at the cell subtype level (right).

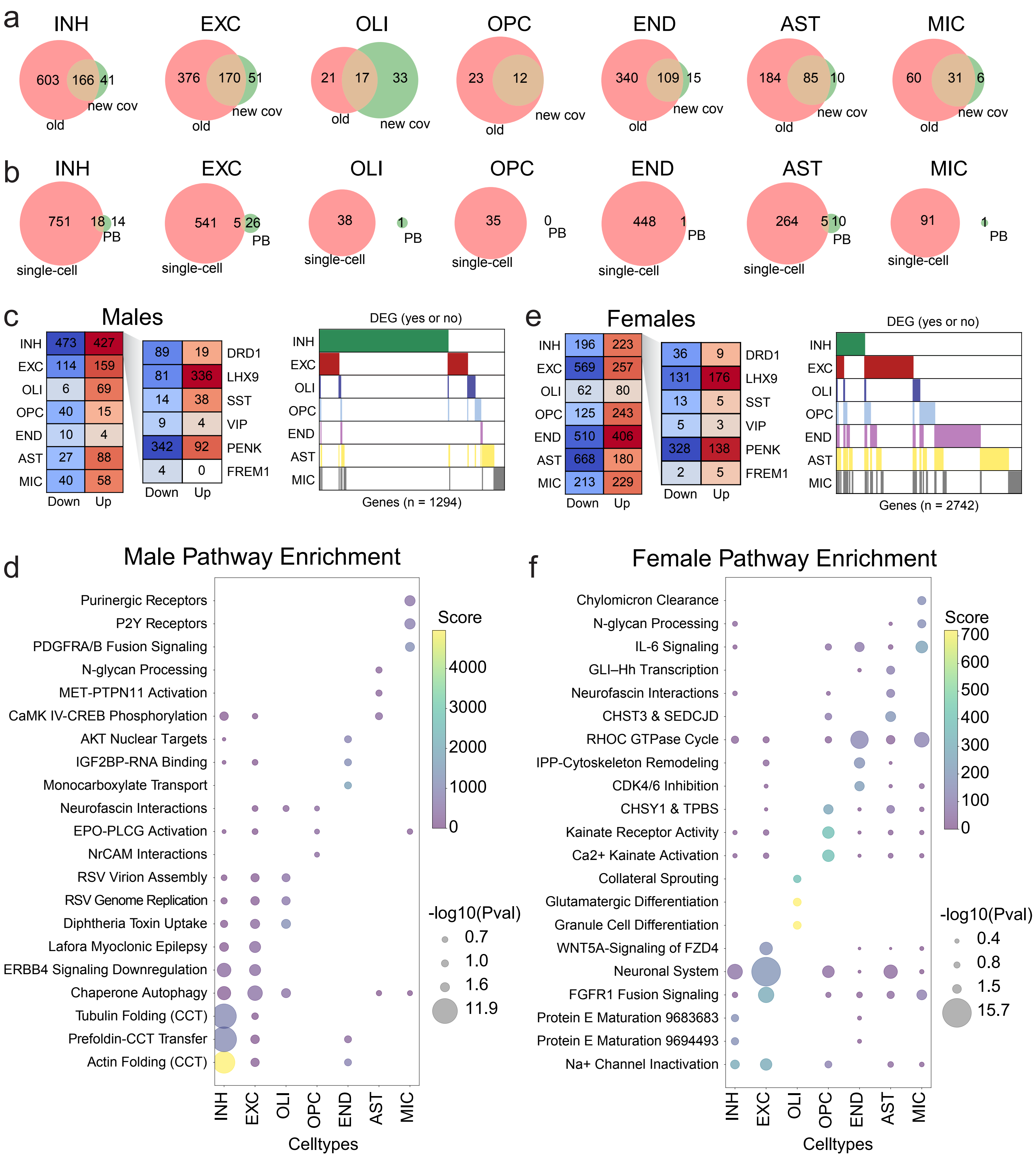
a
INH
603
166
41
old
new cov
EXC
376
170
51
old
new cov
OLI
21
17
33
old
new cov
OPC
23
12
old
new cov
END
340
109
15
old
new cov
AST
184
85
10
old
new cov
MIC
60
31
6
old
new cov
b
INH
751
18
14
single-cell
PB
EXC
541
5
26
single-cell
PB
OLI
38
1
single-cell
PB
OPC
35
0
single-cell
PB
END
448
1
single-cell
PB
AST
264
5
10
single-cell
PB
MIC
91
1
single-cell
PB
c
Males
INH 473 427
EXC 114 159
OLI 6 69
OPC 40 15
END 10 4
AST 27 88
MIC 40 58
Down Up
89 19 DRD1
81 336 LHX9
14 38 SST
9 4 VIP
342 92 PENK
4 0 FREM1
Down Up
DEG (yes or no)
INH
EXC
OLI
OPC
END
AST
MIC
Genes (n = 1294)
e
Females
INH 196 223
EXC 569 257
OLI 62 80
OPC 125 243
END 510 406
AST 668 180
MIC 213 229
Down Up
36 9 DRD1
131 176 LHX9
13 5 SST
5 3 VIP
328 138 PENK
2 5 FREM1
Down Up
DEG (yes or no)
INH
EXC
OLI
OPC
END
AST
MIC
Genes (n = 2742)
d
Male Pathway Enrichment
Purinergic Receptors
P2Y Receptors
PDGFRA/B Fusion Signaling
N-glycan Processing
MET-PTPN11 Activation
CaMK IV-CREB Phosphorylation
AKT Nuclear Targets
IGF2BP-RNA Binding
Monocarboxylate Transport
Neurofascin Interactions
EPO-PLCG Activation
NrCAM Interactions
RSV Virion Assembly
RSV Genome Replication
Diphtheria Toxin Uptake
Lafora Myoclonic Epilepsy
ERBB4 Signaling Downregulation
Chaperone Autophagy
Tubulin Folding (CCT)
Prefoldin-CCT Transfer
Actin Folding (CCT)
INH
EXC
OLI
OPC
END
AST
MIC
Celltypes
Score
4000
3000
2000
1000
0
-log10(Pval)
0.7
1.0
1.6
11.9
f
Female Pathway Enrichment
Chylomicron Clearance
N-glycan Processing
IL-6 Signaling
GLI–Hh Transcription
Neurofascin Interactions
CHST3 & SEDCJD
RHOC GTPase Cycle
IPP-Cytoskeleton Remodeling
CDK4/6 Inhibition
CHSY1 & TPBS
Kainate Receptor Activity
Ca2+ Kainate Activation
Collateral Sprouting
Glutamatergic Differentiation
Granule Cell Differentiation
WNT5A-Signaling of FZD4
Neuronal System
FGFR1 Fusion Signaling
Protein E Maturation 9683683
Protein E Maturation 9694493
Na+ Channel Inactivation
INH
EXC
OLI
OPC
END
AST
MIC
Celltypes
Score
700
600
500
400
300
200
100
0
-log10(Pval)
0.4
0.8
1.5
15.7

Supplementary Figure 6: Covariate, PseudoBulk, Sex-based DEGs. Venn diagram showing overlap of DEGs in Figure 3 with **a,** DEGs with additional clinical covariates and **b,** DEGs from pseudobulk method. Significant DEG counts for males **c,** in both directions for each major cell type (left) and INH sub cell types (right) and **d,** with their top enrichR Reactome (Pathway) terms. Significant DEG counts for females **e,** in both directions for each major cell type (left) and INH sub cell types (right) and **f,** with their top enrichR Reactome (Pathway) terms.

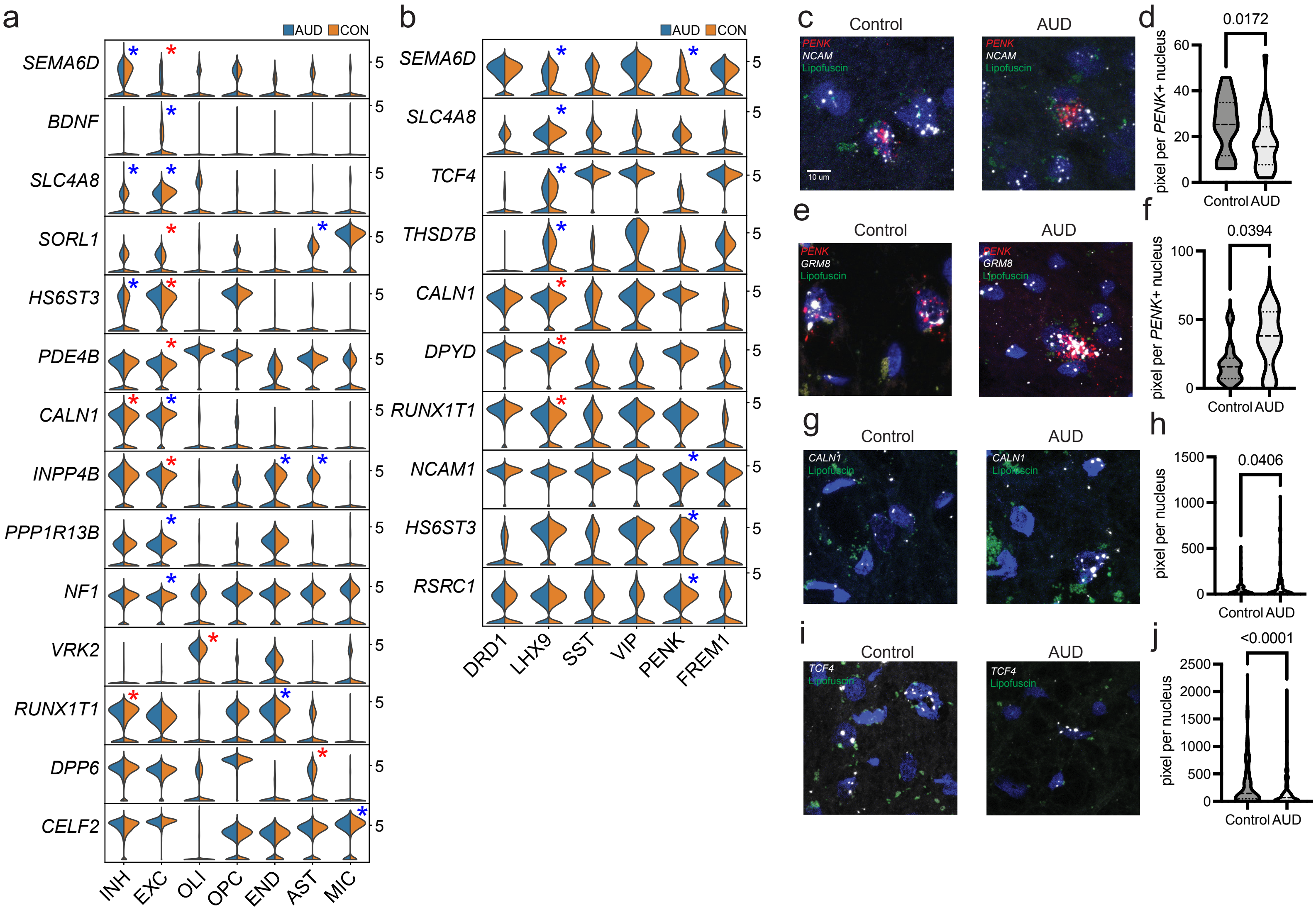

a
AUD
CON
SEMA6D
BDNF
SLC4A8
SORL1
HS6ST3
PDE4B
CALN1
INPP4B
PPP1R13B
NF1
VRK2
RUNX1T1
DPP6
CELF2
INH
EXC
OLI
OPC
END
AST
MIC
b
SEMA6D
SLC4A8
TCF4
THSD7B
CALN1
DPYD
RUNX1T1
NCAM1
HS6ST3
RSRC1
DRD1
LHX9
SST
VIP
PENK
FREM1
c
Control
AUD
PENK
NCAM
Lipofuscin
10 um
d
0.0172
pixel per PENK+ nucleus
Control
AUD
e
Control
AUD
PENK
GRM8
Lipofuscin
f
0.0394
pixel per PENK+ nucleus
g
Control
AUD
CALN1
Lipofuscin
h
0.0406
pixel per nucleus
i
Control
AUD
TCF4
Lipofuscin
j
<0.0001
pixel per nucleus

Supplementary Figure 7: 22 GWAS DEGs. a, Violin plot showing GWAS DEGs in the non-inhibitory cell types with significant up (red asterisk) and down (blue asterisk) expression. **b,** Violin plot showing GWAS DEGs in the INH cell subtypes with significant up (red asterisk) and down (blue asterisk) expression. **c**, Example images of FISH from control (left) and AUD (right) tissue probed for *PENK* (red) and *NCAM1* (white). **d**, Average *NCAM1* pixel number per *PENK*+ nucleus. Mann-Whitney test, Control: n = 24 nuclei, AUD: n = 29 nuclei. **e**, Example images of FISH from control (left) and AUD (right) tissue probed for *PENK* (red) and *GRM8* (white). **f**, Average *GRM8* pixel number per *PENK*+ nucleus. Unpaired t test, Control: n =10 nuclei, AUD: n = 9 nuclei. **g**, Example images of FISH from control (left) and AUD (right) tissue probed for *CALN1* (white). **h**, Average *CALN1* pixel number per nucleus. Unpaired t test, Control: n = 103 nuclei, AUD: n = 91 nuclei. **i**, Example images of FISH from control (left) and AUD (right) tissue probed for *TCF4* (white). **j**, Average *TCF4* pixel number per nucleus. Mann-Whitney test, Control: n = 189 nuclei, AUD: n = 119 nuclei. 3 control donors and 3 AUD donors were used for these analyses. 3 ROIs were analyzed per donor. *P* values indicated on violin plots. Green: Lipofusion. Blue: DAPI.

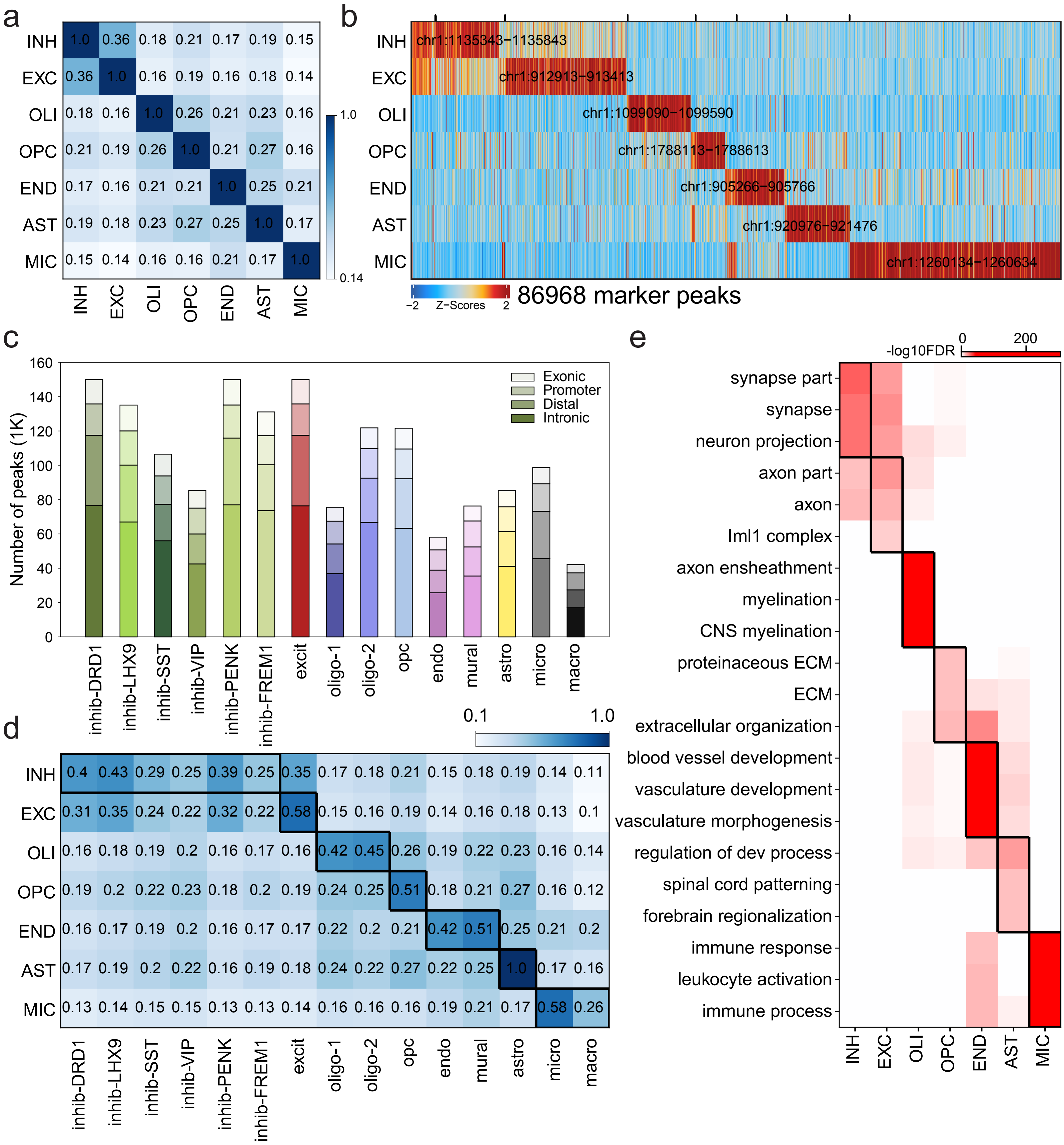
a
INH 1.0 0.36 0.18 0.21 0.17 0.19 0.15
EXC 0.36 1.0 0.16 0.19 0.16 0.18 0.14
OLI 0.18 0.16 1.0 0.26 0.21 0.23 0.16
OPC 0.21 0.19 0.26 1.0 0.21 0.27 0.16
END 0.17 0.16 0.21 0.21 1.0 0.25 0.21
AST 0.19 0.18 0.23 0.27 0.25 1.0 0.17
MIC 0.15 0.14 0.16 0.16 0.21 0.17 1.0
1.0
0.14
INH EXC OLI OPC END AST MIC
b
INH chr1:1135343−1135843
EXC chr1:912913−913413
OLI chr1:1099090−1099590
OPC chr1:1788113−1788613
END chr1:905266−905766
AST chr1:920976−921476
MIC chr1:1260134−1260634
−2 Z−Scores 2
86968 marker peaks
c
Number of peaks (1K)
0 20 40 60 80 100 120 140 160
Exonic
Promoter
Distal
Intronic
inhib-DRD1 inhib-LHX9 inhib-SST inhib-VIP inhib-PENK inhib-FREM1 excit oligo-1 oligo-2 opc endo mural astro micro macro
d
0.1 1.0
INH 0.4 0.43 0.29 0.25 0.39 0.25 0.35 0.17 0.18 0.21 0.15 0.18 0.19 0.14 0.11
EXC 0.31 0.35 0.24 0.22 0.32 0.22 0.58 0.15 0.16 0.19 0.14 0.16 0.18 0.13 0.1
OLI 0.16 0.18 0.19 0.2 0.16 0.17 0.16 0.42 0.45 0.26 0.19 0.22 0.23 0.16 0.14
OPC 0.19 0.2 0.22 0.23 0.18 0.2 0.19 0.24 0.25 0.51 0.18 0.21 0.27 0.16 0.12
END 0.16 0.17 0.19 0.2 0.16 0.17 0.17 0.22 0.2 0.21 0.42 0.51 0.25 0.21 0.2
AST 0.17 0.19 0.2 0.22 0.16 0.19 0.18 0.24 0.22 0.27 0.22 0.25 1.0 0.17 0.16
MIC 0.13 0.14 0.15 0.15 0.13 0.13 0.14 0.16 0.16 0.16 0.19 0.21 0.17 0.58 0.26
inhib-DRD1 inhib-LHX9 inhib-SST inhib-VIP inhib-PENK inhib-FREM1 excit oligo-1 oligo-2 opc endo mural astro micro macro
e
-log10FDR 0 200
synapse part
synapse
neuron projection
axon part
axon
Iml1 complex
axon ensheathment
myelination
CNS myelination
proteinaceous ECM
ECM
extracellular organization
blood vessel development
vasculature development
vasculature morphogenesis
regulation of dev process
spinal cord patterning
forebrain regionalization
immune response
leukocyte activation
immune process
INH EXC OLI OPC END AST MIC

Supplementary Figure 8: ATAC peak features at the celltype and subtype levels. a, Jaccard similarity matrix showing the degree of overlap in peaks across cell types ranging from 0 (no overlap) to 1 (complete overlap). The Jaccard statistic measures the ratio of the number of intersecting base pairs between two sets to the number of base pairs in the union of the two sets. EXC and INH share the highest similarity. **b,** Marker peak heat map showing high degree of cell type specificity in snATAC peaks. The 86,968 peaks shown here are based on thresholds: FDR <= 0.01 and Log2FC >= 0.5. Labeled on the plot is the first of each celltype's marker peaks ordered by genomic region. **c,** Stacked barplot showing number of peaks in each sub celltype (15) separated by genomic category. **d,** Jaccard similarity matrix showing the degree of overlap in peaks between cell types and cell subtypes. **e,** Gene ontology analysis of marker peaks in **b)** using GREAT. GO terms were prioritized based on cell type-specificity.

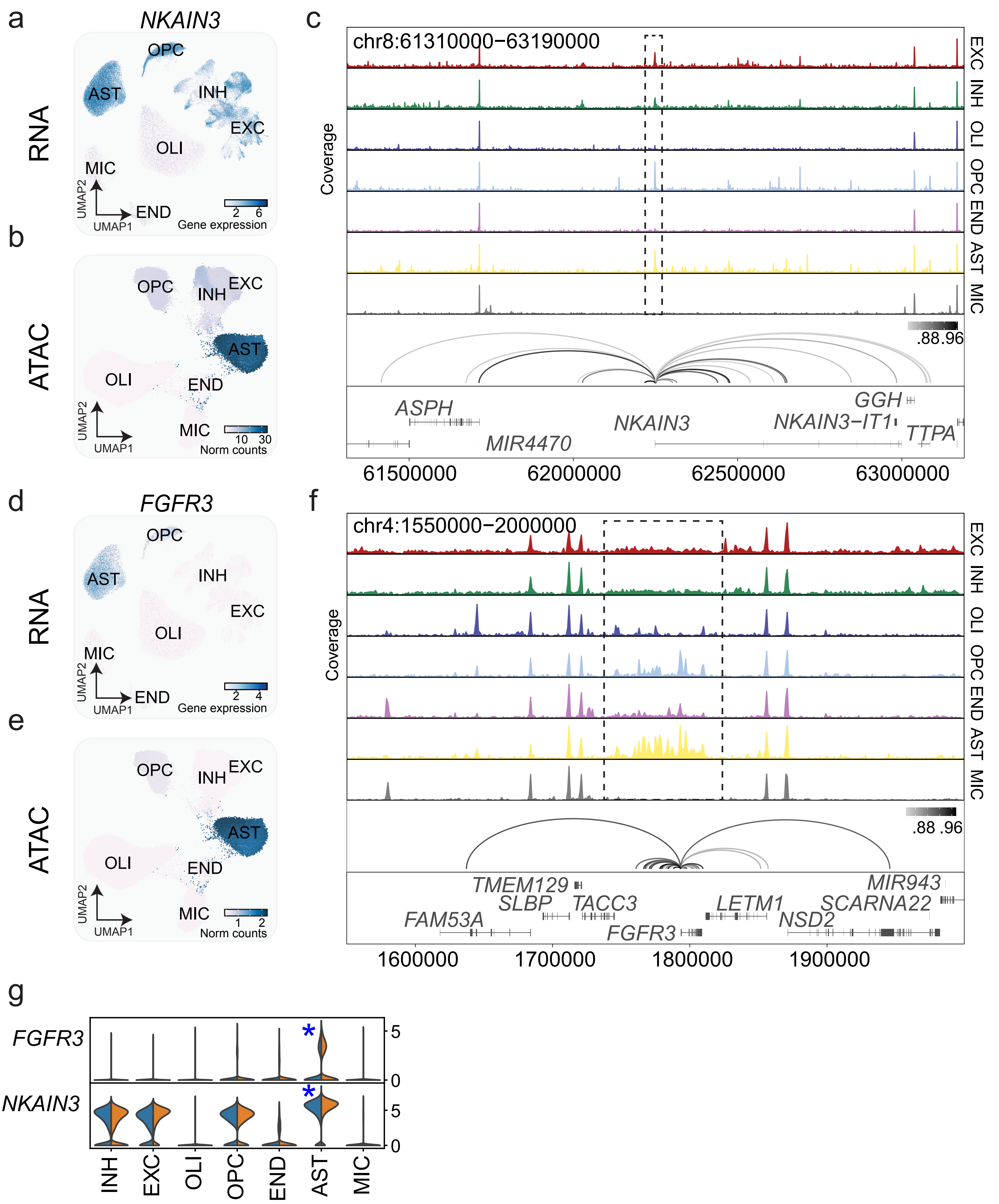
a
NKAIN3
RNA
OPC
AST
INH
EXC
OLI
MIC
END
UMAP2
UMAP1
2 6
Gene expression
b
ATAC
OPC
INH
EXC
AST
OLI
END
MIC
10 30
Norm counts
c
chr8:61310000−63190000
Coverage
EXC
INH
OLI
OPC
END
AST
MIC
.88 .96
ASPH
GGH
NKAIN3
NKAIN3−IT1
TTPA
MIR4470
61500000
62000000
62500000
63000000
d
FGFR3
2 4
Gene expression
e
1 2
Norm counts
f
chr4:1550000−2000000
TMEM129
SLBP
TACC3
LETM1
MIR943
SCARNA22
FAM53A
NSD2
FGFR3
1600000
1700000
1800000
1900000
g
FGFR3
NKAIN3
*
5
0
INH
EXC
OLI
OPC
END
AST
MIC

Supplementary Figure 9: Astrocytes multi-omic profile. UMAP plots of *NKAIN3* snRNA-seq gene expression on the **a,** snRNA (top) and **b,** snATAC-seq (bottom) embeddings. **c,** Chromatin accessibility signal tracks highlighting *NKAIN3* peak-to-gene links across cell types. Differences across the seven cell types are indicated by the dashed boxes. UMAP plots of *FGFR3* snRNA-seq gene expression on the **d,** snRNA (top) and **e,** snATAC-seq (bottom) embeddings. **f,** Chromatin accessibility signal tracks highlighting *FGFR3* peak-to-gene links across cell types. Differences across the seven cell types are indicated by the dashed boxes. **g,** Violin plot showing AUD versus CON expression of *FGFR3*, *NKAIN3* in each cell type with significant up (red asterisk) and down (blue asterisk) expression.

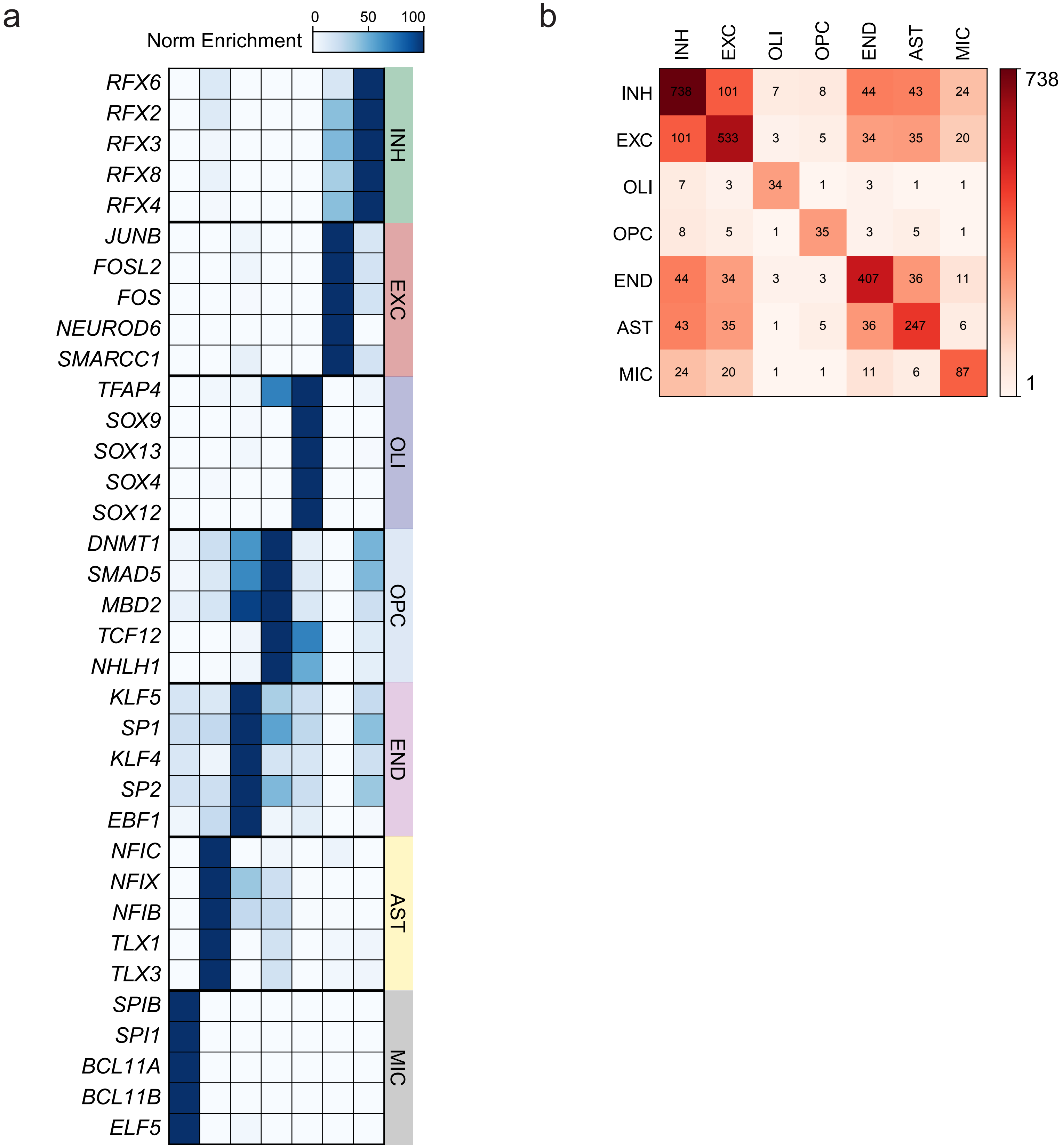
a
Norm Enrichment
0
50
100
RFX6
RFX2
RFX3
RFX8
RFX4
JUNB
FOSL2
FOS
NEUROD6
SMARCC1
TFAP4
SOX9
SOX13
SOX4
SOX12
DNMT1
SMAD5
MBD2
TCF12
NHLH1
KLF5
SP1
KLF4
SP2
EBF1
NFIC
NFIX
NFIB
TLX1
TLX3
SPIB
SPI1
BCL11A
BCL11B
ELF5
INH
EXC
OLI
OPC
END
AST
MIC
b
INH EXC OLI OPC END AST MIC
INH 738 101 7 8 44 43 24
EXC 101 533 3 5 34 35 20
OLI 7 3 34 1 3 1 1
OPC 8 5 1 35 3 5 1
END 44 34 3 3 407 36 11
AST 43 35 1 5 36 247 6
MIC 24 20 1 1 11 6 87
738
1

Supplementary Figure 10: GRN network construction. a, TF motif heatmap of highly enriched TFs for each cell type. **b,** Matrix showing overlap of CRE-linked-DEGs across cell types. EXN and INH have the highest overlap.

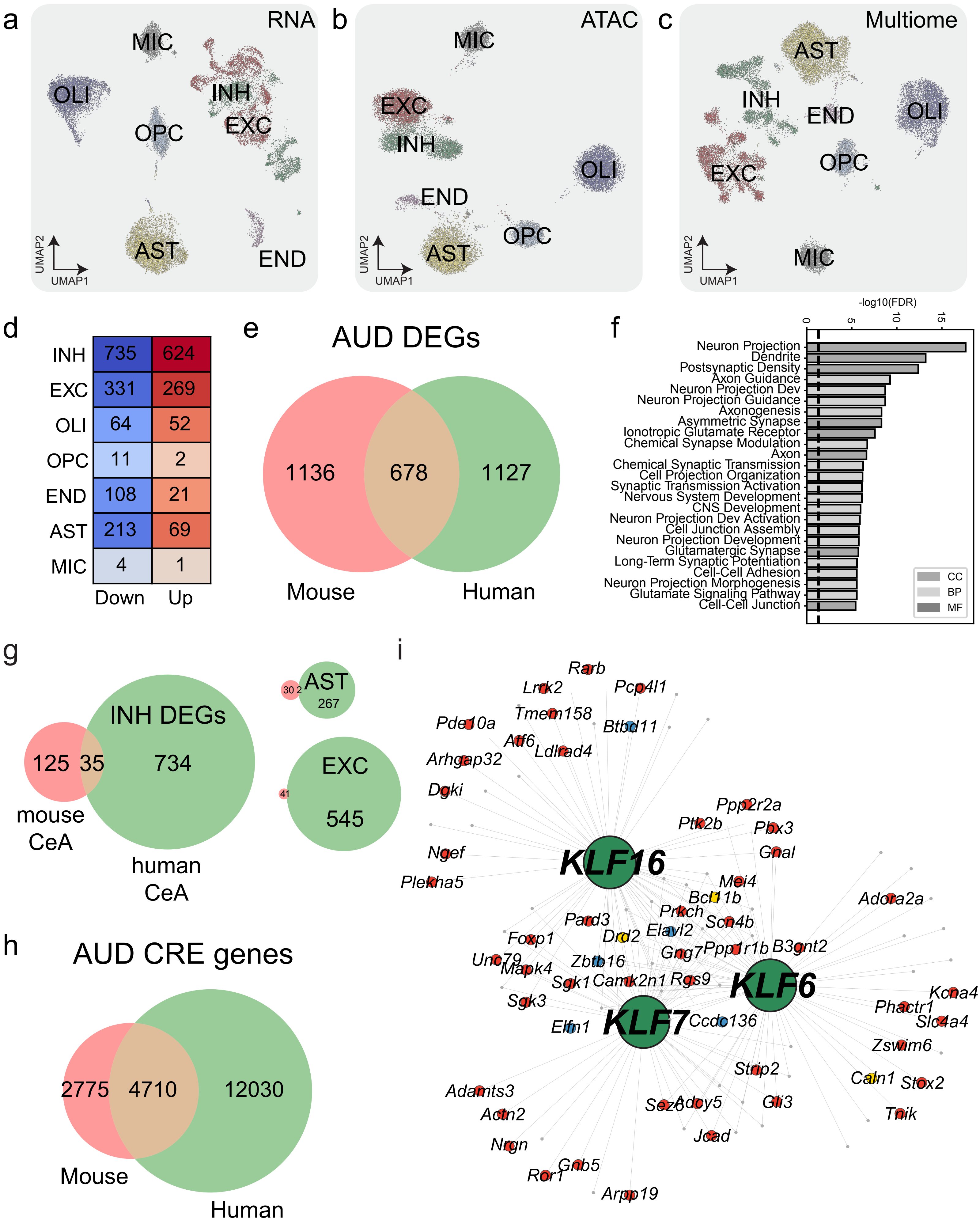

a
RNA
MIC
OLI
INH
OPC
EXC
AST
END
UMAP2
UMAP1
b
ATAC
MIC
EXC
INH
OLI
END
OPC
AST
c
Multiome
AST
INH
END
OLI
OPC
EXC
MIC
d
INH 735 624
EXC 331 269
OLI 64 52
OPC 11 2
END 108 21
AST 213 69
MIC 4 1
Down Up
e
AUD DEGs
1136
678
1127
Mouse
Human
f
-log10(FDR)
0 5 10 15
Neuron Projection
Dendrite
Postsynaptic Density
Axon Guidance
Neuron Projection Dev
Neuron Projection Guidance
Axonogenesis
Asymmetric Synapse
Ionotropic Glutamate Receptor
Chemical Synapse Modulation
Axon
Chemical Synaptic Transmission
Cell Projection Organization
Synaptic Transmission Activation
Nervous System Development
CNS Development
Neuron Projection Dev Activation
Cell Junction Assembly
Neuron Projection Development
Glutamatergic Synapse
Long-Term Synaptic Potentiation
Cell-Cell Adhesion
Neuron Projection Morphogenesis
Glutamate Signaling Pathway
Cell-Cell Junction
CC
BP
MF
g
INH DEGs
125 35
734
mouse CeA
human CeA
30 2
AST
267
EXC
41
545
h
AUD CRE genes
2775
4710
12030
Mouse
Human
i
KLF16
KLF6
KLF7
Rarb
Lrrk2
Pcp4l1
Tmem158
Btbd11
Pde10a
Atf6
Ldlrad4
Arhgap32
Dgki
Ppp2r2a
Ptk2b
Pbx3
Ngef
Gnal
Plekha5
Mei4
Bcl11b
Adora2a
Prkch
Pard3
Scn4b
Foxp1
Drd2
Elavl2
Unc79
Gng7
Ppp1r1b
B3gnt2
Mapk4
Zbtb16
Sgk1
Camk2n1
Rgs9
Kcna4
Sgk3
Phactr1
Elfn1
Ccdc136
Slc4a4
Zswim6
Strip2
Caln1
Stox2
Adamts3
Sez6
Adcy5
Gli3
Tnik
Actn2
Jcad
Nrgn
Gnb5
Rorl
Arpp19

Supplementary Figure 11: Mouse snMultiome data. UMAP visualization across seven cell types of **a,** snRNA-seq **b,** snATAC-seq across seven cell types and **c,** snMultiome. **d,** Significant DEG counts in both directions for each major cell type. **e,** Intersection of AUD DEGs between human and mouse. **f,** Top 25 enrichR GO terms of the 1,814 unique AUD DEG set. **g,** Intersection of alcohol DEGs between human and mouse central amygdala from literature[50]. **h,** Intersection of alcohol Cis-Regulatory Element–linked genes between human and mouse. **i,** TF regulatory network showing the target genes for TFs *KLF6*, *KLF7*, and *KLF16* in INH. Peak-to-gene correlation > 0.9 was employed in visualizing the network.

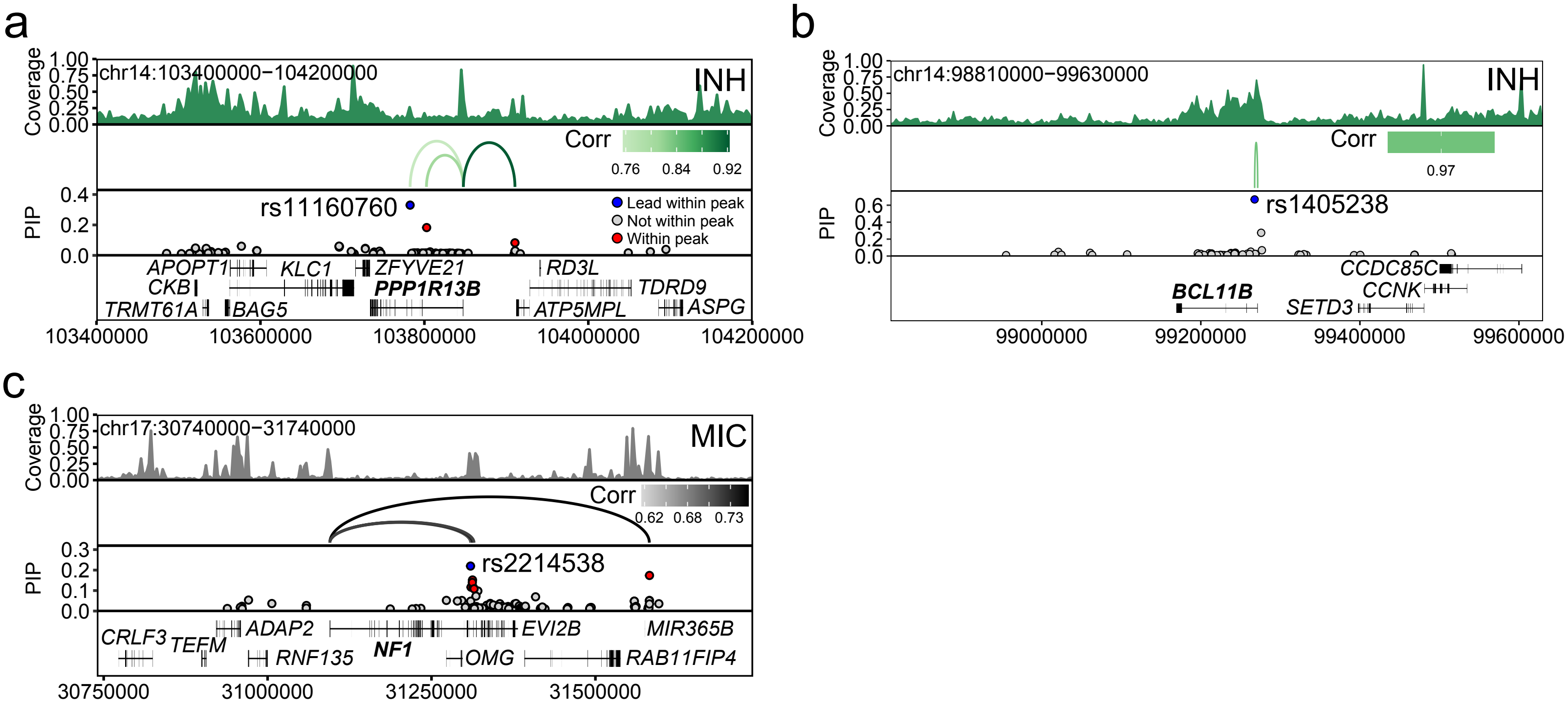
a
Coverage
chr14:103400000−104200000
INH
Corr
0.76 0.84 0.92
PIP
rs11160760
Lead within peak
Not within peak
Within peak
APOPT1
KLC1
ZFYVE21
RD3L
CKB
PPP1R13B
TDRD9
TRMT61A
BAG5
ATP5MPL
ASPG
103400000 103600000 103800000 104000000 104200000
b
chr14:98810000−99630000
INH
Corr
0.97
rs1405238
CCDC85C
BCL11B
CCNK
SETD3
99000000 99200000 99400000 99600000
c
chr17:30740000−31740000
MIC
Corr
0.62 0.68 0.73
rs2214538
CRLF3
TEFM
ADAP2
NF1
EVI2B
MIR365B
RNF135
OMG
RAB11FIP4
30750000 31000000 31250000 31500000

Supplementary Figure 12: PAU risk SNP Fine-mapping. **a**, *Cis*-regulatory architecture for *PPP1R13B* in INH for PAU EUR GWAS (chr14:103400000−104200000). **b,** *Cis*-regulatory architecture for *BCL11B* in INH for PAU EUR GWAS (chr14:98810000−99630000). **c,** *Cis*-regulatory architecture for *NF1* in MIC for PAU EUR GWAS (chr17:30740000−31740000).

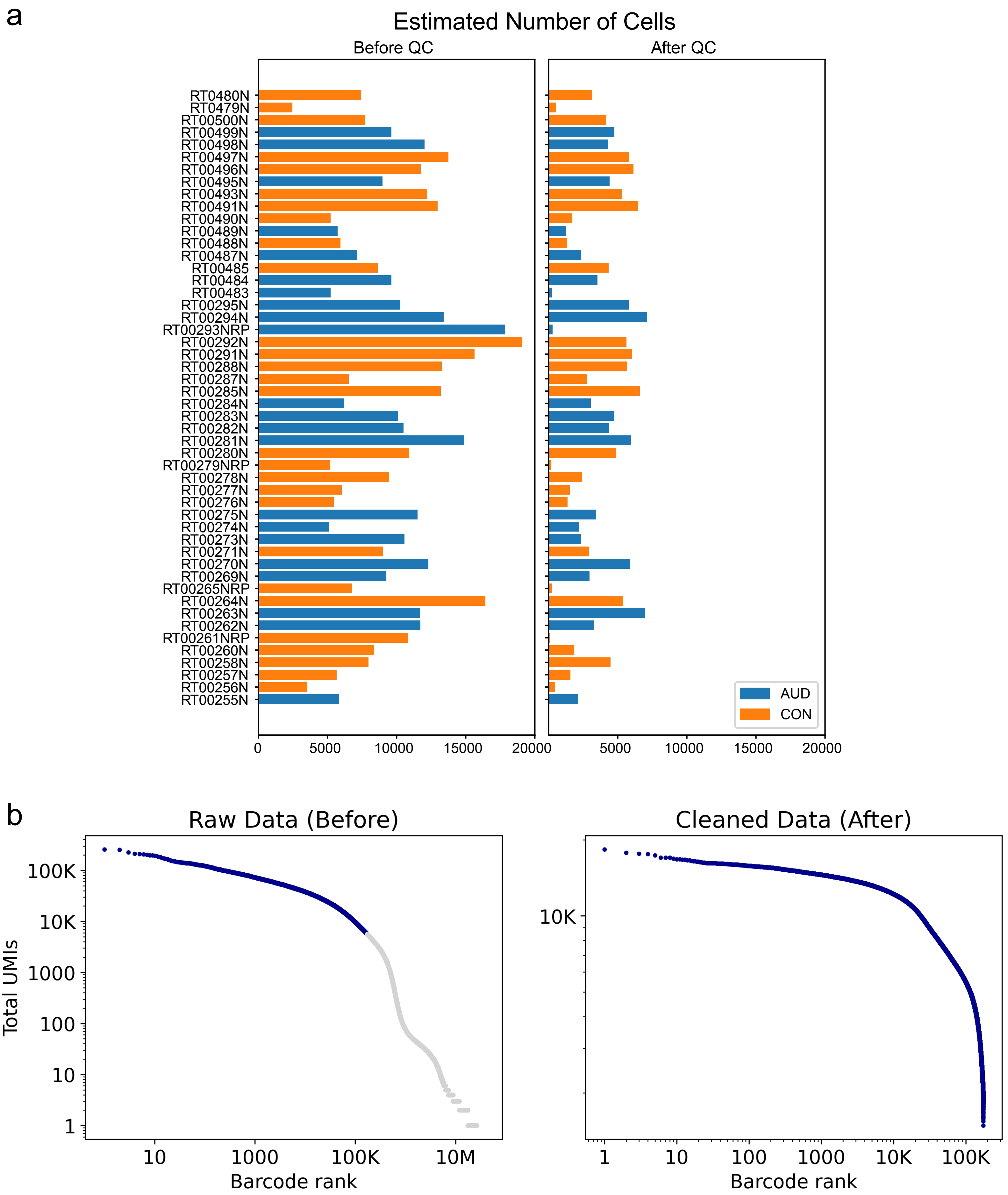
a
Estimated Number of Cells
Before QC
After QC
RT0480N
RT0479N
RT00500N
RT00499N
RT00498N
RT00497N
RT00496N
RT00495N
RT00493N
RT00491N
RT00490N
RT00489N
RT00488N
RT00487N
RT00485
RT00484
RT00483
RT00295N
RT00294N
RT00293NRP
RT00292N
RT00291N
RT00288N
RT00287N
RT00285N
RT00284N
RT00283N
RT00282N
RT00281N
RT00280N
RT00279NRP
RT00278N
RT00277N
RT00276N
RT00275N
RT00274N
RT00273N
RT00271N
RT00270N
RT00269N
RT00265NRP
RT00264N
RT00263N
RT00262N
RT00261NRP
RT00260N
RT00258N
RT00257N
RT00256N
RT00255N
AUD
CON
0
5000
10000
15000
20000
5000
10000
15000
20000
b
Raw Data (Before)
Cleaned Data (After)
Total UMIs
100K
10K
1000
100
10
1
10
1000
100K
10M
Barcode rank
10K
1
10
100
1000
10K
100K
Barcode rank

Supplementary Figure 13: Sample quality control. a, Sample cell count before and after quality control. **b,** Barcode-rank plot across all samples.

Supplementary Data

Supplementary Data 1: Samples Demographic Metadata

Supplementary Data 2: Differentially Expressed Genes with MAST and Wilcox intersection

Supplementary Data 3: Differentially Expressed Genes from MAST

Supplementary Data 4: Differentially Expressed Genes from Wilcox

Supplementary Data 5: Differentially Expressed Genes with MAST and Wilcox intersection passing the Fold Change and FDR thresholds

Supplementary Data 6: Differentially Expressed Genes with MAST and Wilcox intersection passing the Fold Change and FDR thresholds, with Clinical Covariates

Supplementary Data 7: Differentially Expressed Genes from Pseudobulk Analysis with multiple comparison-corrected two-sided Wald's test

Supplementary Data 8: Sex-based Differentially Expressed Genes in Males

Supplementary Data 9: Sex-based Differentially Expressed Genes in Females

Supplementary Data 10: Sex-based Differentially Expressed Genes between Males and Females in CON condition

Supplementary Data 11: Gene Ontology of all significant DEGs by two-sided Fisher exact test with corrections for multiple comparisons

Supplementary Data 12: Gene Ontology of all significant DEGs categorized by cell types by two-sided Fisher exact test with corrections for multiple comparisons

Supplementary Data 13: Gene Ontology of all significant DEGs categorized by cell types and Reactome Pathways by two-sided Fisher exact test with corrections for multiple comparisons

Supplementary Data 14: snATAC Peaks

Supplementary Data 15: snATAC Union Peaks

Supplementary Data 16: snATAC Union Cis-Regulatory Element Peaks

Supplementary Data 17: AUD Disease-specific INH Peaks

Supplementary Data 18: AUD Disease-specific EXC Peaks

Supplementary Data 19: AUD Disease-specific OLI Peaks

Supplementary Data 20: AUD Disease-specific OPC Peaks

Supplementary Data 21: AUD Disease-specific END Peaks

Supplementary Data 22: AUD Disease-specific AST Peaks

Supplementary Data 23: AUD Disease-specific MIC Peaks

Supplementary Data 24: Gene Regulatory Network for INH

Supplementary Data 25: Gene Regulatory Network for EXC

Supplementary Data 26: Gene Regulatory Network for OLI

Supplementary Data 27: Gene Regulatory Network for OPC

Supplementary Data 28: Gene Regulatory Network for END

Supplementary Data 29: Gene Regulatory Network for AST

Supplementary Data 30: Gene Regulatory Network for MIC

Supplementary Data 31: Transcription Factors with number of linked DEG and GWAS genes

Supplementary Data 32: Gene Regulatory Network for INH, for the KLF 6, 7, 16 Transcription Factors

Supplementary Data 33: Linkage Disequilibrium Score Regression analysis data for CeA cell type-specific Peaks, by two-sided z-test with adjustments for multiple comparisons

Supplementary Data 34: Linkage Disequilibrium Score Regression analysis data for INH Peaktype-specific Peaks, by two-sided z-test

Supplementary Data 35: Finemapping SNPs discovered with snATAC data as prior weights

Supplementary Data 36: Finemapping SNPs confirmed with prior AUD GWAS study

Supplementary Data 37: Samples Cell Count Metadata
