# Supplementary material for: Central amygdala single-nucleus atlas reveals chromatin and gene transcription dynamics in human alcohol use disorder
Source: Nat Commun. 2026 Jan 19;17:1634. doi: 10.1038/s41467-026-68351-1 (PMC12905162; doi:10.1038/s41467-026-68351-1)
Supplement: Supplementary file 2 — Description of Additional Supplementary Files [file 41467_2026_68351_MOESM2_ESM.pdf]

Description of Additional Supplementary Files

File Name: Supplementary Data 1
Description: Sample Demographic Metadata

File Name: Supplementary Data 2
Description: Differentially Expressed Genes with MAST and Wilcox intersection

File Name: Supplementary Data 3
Description: Differentially Expressed Genes from MAST

File Name: Supplementary Data 4
Description: Differentially Expressed Genes from Wilcox

File Name: Supplementary Data 5
Description: Differentially Expressed Genes with MAST and Wilcox intersection passing the Fold Change and FDR thresholds

File Name: Supplementary Data 6
Description: Differentially Expressed Genes with MAST and Wilcox intersection passing the Fold Change and FDR thresholds, with Clinical Covariates

File Name: Supplementary Data 7
Description: Differentially Expressed Genes from Pseudobulk Analysis with multiple comparison-corrected two-sided Wald's test

File Name: Supplementary Data 8
Description: Sex-specific Differentially Expressed Genes in Males

File Name: Supplementary Data 9
Description: Sex-specific Differentially Expressed Genes in Females

File Name: Supplementary Data 10
Description: Basline Differentially Expressed Genes between Males and Females in controls

File Name: Supplementary Data 11
Description: Gene Ontology of all significant DEGs by two-sided Fisher exact test with corrections for multiple comparisons

File Name: Supplementary Data 12
Description: Gene Ontology of all significant DEGs categorized by cell type by two-sided Fisher exact test with corrections for multiple comparisons

File Name: Supplementary Data 13
Description: Gene Ontology of all significant DEGs categorized by cell type and Reactome Pathways by two-sided Fisher exact test with corrections for multiple comparisons

File Name: Supplementary Data 14
Description: snATAC Peaks

File Name: Supplementary Data 15
Description: snATAC Union Peaks

File Name: Supplementary Data 16
Description: snATAC Union Cis-Regulatory Element Peaks

File Name: Supplementary Data 17
Description: AUD Disease-specific INH Peaks

File Name: Supplementary Data 18
Description: AUD Disease-specific EXC Peaks

File Name: Supplementary Data 19
Description: AUD Disease-specific OLI Peaks

File Name: Supplementary Data 20
Description: AUD Disease-specific OPC Peaks

File Name: Supplementary Data 21
Description: AUD Disease-specific END Peaks

File Name: Supplementary Data 22
Description: AUD Disease-specific AST Peaks

File Name: Supplementary Data 23
Description: AUD Disease-specific MIC Peaks

File Name: Supplementary Data 24
Description: Gene Regulatory Network for INH

File Name: Supplementary Data 25
Description: Gene Regulatory Network for EXC

File Name: Supplementary Data 26
Description: Gene Regulatory Network for OLI

File Name: Supplementary Data 27
Description: Gene Regulatory Network for OPC

File Name: Supplementary Data 28
Description: Gene Regulatory Network for END

File Name: Supplementary Data 29
Description: Gene Regulatory Network for AST

File Name: Supplementary Data 30
Description: Gene Regulatory Network for MIC

File Name: Supplementary Data 31
Description: Transcription Factors with number of linked DEG and GWAS genes

File Name: Supplementary Data 32
Description: Gene Regulatory Network for INH, for the KLF 6, 7, 16 Transcription Factors

File Name: Supplementary Data 33
Description: Linkage Disequilibrium Score Regression analysis data for CeA cell type-specific Peaks, by two-sided z-test with adjustments for multiple comparisons

File Name: Supplementary Data 34
Description: Linkage Disequilibrium Score Regression analysis data for INH Peaktype-specific Peaks, by two-sided z-test

File Name: Supplementary Data 35
Description: Finemapped SNPs discovered with snATAC data as prior weights

File Name: Supplementary Data 36
Description: Finemapped SNPs confirmed with prior AUD GWAS study

File Name: Supplementary Data 37
Description: Samples Cell Count Metadata
