# Supplementary material for: Central amygdala single-nucleus atlas reveals chromatin and gene transcription dynamics in human alcohol use disorder
Source: Nat Commun. 2026 Jan 19;17:1634. doi: 10.1038/s41467-026-68351-1 (PMC12905162; doi:10.1038/s41467-026-68351-1)
Supplement: Supplementary file 4 — Reporting Summary [file 41467_2026_68351_MOESM4_ESM.pdf]

nature portfolio

Corresponding author(s): Matthew Girgenti, PhD
Jing Zhang, PhD

Last updated by author(s): Nov 18, 2025

Reporting Summary

Nature Portfolio wishes to improve the reproducibility of the work that we publish. This form provides structure for consistency and transparency in reporting. For further information on Nature Portfolio policies, see our Editorial Policies and the Editorial Policy Checklist.

Statistics

For all statistical analyses, confirm that the following items are present in the figure legend, table legend, main text, or Methods section.

n/a	Confirmed	
☐	☒	The exact sample size (*n*) for each experimental group/condition, given as a discrete number and unit of measurement
☐	☒	A statement on whether measurements were taken from distinct samples or whether the same sample was measured repeatedly
☐	☒	The statistical test(s) used AND whether they are one- or two-sided<br>*Only common tests should be described solely by name; describe more complex techniques in the Methods section.*
☐	☒	A description of all covariates tested
☐	☒	A description of any assumptions or corrections, such as tests of normality and adjustment for multiple comparisons
☐	☒	A full description of the statistical parameters including central tendency (e.g. means) or other basic estimates (e.g. regression coefficient) AND variation (e.g. standard deviation) or associated estimates of uncertainty (e.g. confidence intervals)
☐	☒	For null hypothesis testing, the test statistic (e.g. *F*, *t*, *r*) with confidence intervals, effect sizes, degrees of freedom and *P* value noted<br>*Give P values as exact values whenever suitable.*
☒	☐	For Bayesian analysis, information on the choice of priors and Markov chain Monte Carlo settings
☐	☒	For hierarchical and complex designs, identification of the appropriate level for tests and full reporting of outcomes
☐	☒	Estimates of effect sizes (e.g. Cohen's *d*, Pearson's *r*), indicating how they were calculated

Our web collection on statistics for biologists contains articles on many of the points above.

Software and code

Policy information about availability of computer code

Data collection	No software was used for Data collection
Data analysis	All software used for data analysis is available on the GitHub: https://github.com/mjgirgenti/AUDsnCEA

For manuscripts utilizing custom algorithms or software that are central to the research but not yet described in published literature, software must be made available to editors and reviewers. We strongly encourage code deposition in a community repository (e.g. GitHub). See the Nature Portfolio guidelines for submitting code & software for further information.

Data

Policy information about availability of data

All manuscripts must include a data availability statement. This statement should provide the following information, where applicable:

- Accession codes, unique identifiers, or web links for publicly available datasets
- A description of any restrictions on data availability
- For clinical datasets or third party data, please ensure that the statement adheres to our policy

The snMultiome data generated in this study have been deposited in the Zenodo database [https://doi.org/10.5281/zenodo.17656668]. The raw data are available through a data use agreement with the National PTSD Brain Bank. Interested investigators should submit dataset requests to the corresponding author and https://www.research.va.gov/programs/tissue_banking/ptsd/ and reference this paper for more information. The processed data generated in this study are provided in the Supplementary Information/Source Data file. Source data are provided with this paper.

Research involving human participants, their data, or biological material

Policy information about studies with human participants or human data. See also policy information about sex, gender (identity/presentation), and sexual orientation and race, ethnicity and racism.

Reporting on sex and gender	Our study was evenly powered for males and females across all -omics data including snRNA, snATAC and snMultiome sequencing. Sex-specific findings are reported in the manuscript
Reporting on race, ethnicity, or other socially relevant groupings	>90% of our cohort is Caucasian and the remaining are African, Asian and Hispanic American. All demographics for ancestry are included in Supplementary Figure 1.
Population characteristics	This group includes donors with alcohol use disoder and neurotypical controls with no history of psychiatric disorder. Full demographics are provided in Extended Data Figure 1 includes demographics for all donors. All analyses included covariate correction for common confounders (see Methods). The list of covariates are sex, ancestry, age, PMI, RIN, smoking, and antidepressant usage.
Recruitment	N/A
Ethics oversight	Yale University

Note that full information on the approval of the study protocol must also be provided in the manuscript.

Field-specific reporting

Please select the one below that is the best fit for your research. If you are not sure, read the appropriate sections before making your selection.

☒ Life sciences ☐ Behavioural & social sciences ☐ Ecological, evolutionary & environmental sciences

For a reference copy of the document with all sections, see nature.com/documents/nr-reporting-summary-flat.pdf

Life sciences study design

All studies must disclose on these points even when the disclosure is negative.

Sample size	While difficult to predict power in these studies when effect sizes are indeterminate, we stress that our analyses to find changes in DEGs and ATAC peaks typically work well with > 20 samples. There are ~ 25 donors across each cohort.
Data exclusions	15 multiome samples were excluded due to failed quality control.
Replication	There are currently no other sources of AUD postmortem tissue and thus a replication cohort was not possible to include in the current study.
Randomization	Samples were organized by diagnosis: AUD or health control. Relevant covariates for postmortem genomics studies such as sex, ancestry, age, PMI, RIN, smoking and antidepressant use were corrected for.
Blinding	Data collection were performed blind to tissue of origin and randomization was performed at the library preparation and sequencing stage.

Reporting for specific materials, systems and methods

We require information from authors about some types of materials, experimental systems and methods used in many studies. Here, indicate whether each material, system or method listed is relevant to your study. If you are not sure if a list item applies to your research, read the appropriate section before selecting a response.

Materials & experimental systems

n/a	Involved in the study	
☐	☒	Antibodies
☒	☐	Eukaryotic cell lines
☒	☐	Palaeontology and archaeology
☐	☒	Animals and other organisms
☒	☐	Clinical data
☒	☐	Dual use research of concern
☒	☐	Plants

Methods

n/a	Involved in the study	
☒	☐	ChIP-seq
☒	☐	Flow cytometry
☒	☐	MRI-based neuroimaging

Antibodies

Antibodies used	anti-Gad1 R&D Systems AF2086 1:100 dilution<br>anti-PENK Atlas Antibodies HPA013138 1:500 dilution

	anti- FREM1 Proteintech 13086-1-AP 1:50 dilution
Validation	Antibodies have been validated by the respective manufacturer and was also validated by many publications that used them as cited on the manufacturers websites:<br>https://www.rndsystems.com/products/human-mouse-rat-gad1-gad67-antibody_af2086?gclsrc=aw.ds&gad_source=1&gad_campaignid=20164788196&gbraid=0AAAAAD_kmX3O0p6c6Tlc59oacTvSXKzuh&gclid=CjwKCAiAz_DIBhBJEiwAVH2XwBMmlE33uxWuVb_UHsY8AAzQYOUVzI-YfM62gGSUg7T0rFoWv4m8IhoCuAAQAvD_BwE<br><br>https://www.atlasantibodies.com/products/primary-antibodies/triple-a-polyclonals/anti-penk-antibody-hpa013138/<br><br>https://www.ptglab.com/products/FREM1-Antibody-13086-1-AP.htm?srsltid=AfmBOoqmAykckH-ZPrfy3reuAl_RQGZxBcO2Gef56sEx1ZSL8E4UbDzI

Animals and other research organisms

Policy information about studies involving animals; ARRIVE guidelines recommended for reporting animal research, and Sex and Gender in Research

Laboratory animals	mus musculus, C57BL/6 wild type, 2 months old, Until treatment, all mice were group housed and maintained in standard environmental conditions (23 °C; 12 h–12 h light–dark cycle) with ad libitum food and water.
Wild animals	N/A
Reporting on sex	males and females were used. N= 4 males and 4 females. no sex based analyses were performed
Field-collected samples	N/A
Ethics oversight	Institutional Animal Care and Use Committee at Yale University School of Medicine and Yale Animal Resources Center

Note that full information on the approval of the study protocol must also be provided in the manuscript.

Plants

Seed stocks	N/A
Novel plant genotypes	N/A
Authentication	N/A
